# Lung ultrasound score to determine the effect of fraction inspired oxygen during alveolar recruitment on absorption atelectasis in laparoscopic surgery: a randomized controlled trial

**DOI:** 10.1186/s12871-020-01090-y

**Published:** 2020-07-18

**Authors:** Bo Rim Kim, Seohee Lee, Hansu Bae, Minkyoo Lee, Jae-Hyon Bahk, Susie Yoon

**Affiliations:** Department of Anesthesiology and Pain Medicine, Seoul National University Hospital, Seoul National University College of Medicine, 101 Daehak-ro, Jongno-gu, Seoul, 03080 Republic of Korea

**Keywords:** Alveolar recruitment, Lung, Oxygen, Pulmonary atelectasis, Ultrasonography

## Abstract

**Background:**

Although the intraoperative alveolar recruitment maneuver (RM) efficiently treats atelectasis, the effect of Fio_2_ on atelectasis during RM is uncertain. We hypothesized that a high Fio_2_ (1.0) during RM would lead to a higher degree of postoperative atelectasis without benefiting oxygenation when compared to low Fio_2_ (0.4).

**Methods:**

In this randomized controlled trial, patients undergoing elective laparoscopic surgery in the Trendelenburg position were allocated to low- (Fio_2_ 0.4, *n* = 44) and high-Fio_2_ (Fio_2_ 1.0, *n* = 46) groups. RM was performed 1-min post tracheal intubation and post changes in supine and Trendelenburg positions during surgery. We set the intraoperative Fio_2_ at 0.4 for both groups and calculated the modified lung ultrasound score (LUSS) to assess lung aeration after anesthesia induction and at surgery completion. The primary outcome was modified LUSS at the end of the surgery. The secondary outcomes were the intra- and postoperative Pao_2_ to Fio_2_ ratio and postoperative pulmonary complications.

**Results:**

The modified LUSS before capnoperitoneum and RM (*P* = 0.747) were similar in both groups. However, the postoperative modified LUSS was significantly lower in the low Fio_2_ group (median difference 5.0, 95% CI 3.0–7.0, *P* < 0.001). Postoperatively, substantial atelectasis was more common in the high-Fio_2_ group (relative risk 1.77, 95% CI 1.27–2.47, *P* < 0.001). Intra- and postoperative Pao_2_ to Fio_2_ were similar with no postoperative pulmonary complications. Atelectasis occurred more frequently when RM was performed with high than with low Fio_2_; oxygenation was not benefitted by a high-Fio_2._

**Conclusions:**

In patients undergoing laparoscopic surgery in the Trendelenburg position, absorption atelectasis occurred more frequently with high rather than low Fio_2_. No oxygenation benefit was observed in the high-Fio_2_ group.

**Trial registration:**

ClinicalTrials.gov, NCT03943433. Registered 7 May 2019,

## Background

During general anesthesia, atelectasis reportedly occurs in most patients [1], typically due to absorption of gas, compression of the lung tissue, and impairment of surfactant function [2]. Additionally, during laparoscopic surgery, the increased abdominal pressure of capnoperitoneum may shift the diaphragm cranially and decrease respiratory compliance [3, 4]. Compression of basal lung regions due to a stiffened diaphragm would accelerate the formation of atelectasis that was already initiated during anesthesia induction [4]. Moreover, the steep Trendelenburg position used in laparoscopic gynecologic or colon surgery causes the abdominal contents to push the diaphragm more cephalad, resulting in aggravated lung collapse and decreased functional residual capacity [5, 6].

Intraoperative atelectasis is associated with decreased lung compliance, impaired oxygenation, increased pulmonary vascular resistance, and lung injury [5, 7]. Moreover, atelectasis can persist postoperatively and result in respiratory complications, such as hypoxemia and infection, significantly impacting patient recovery [5, 8].

The alveolar recruitment maneuver (RM) with positive-end expiratory pressure (PEEP) has been advocated as efficient for atelectasis treatment [9–13]. Reports on the impact of Fio_2_ during RM on atelectasis development are rare, and have not limited Fio_2_ to the RM per se [14]. While RM with high Fio_2_ can improve oxygenation rapidly, there is a greater possibility of absorption atelectasis occurring.

Although computed tomography has been considered as the gold standard for lung imaging, it is less optimal for routine examination of perioperative atelectasis due to the cumbersomeness and the risk of radiation exposure. On the other hand, lung ultrasound is a portable, non-invasive, and radiation-free device [15, 16]. Recent studies have shown the utility of the lung ultrasound score (LUSS) in the operating room [15–19]. The diagnostic reliability of LUSS for detecting perioperative atelectasis has been verified against computed tomography or magnetic resonance imaging [15, 19].

We prospectively assessed the impact of Fio_2_, specifically during RM, on atelectasis development, using the LUSS. We hypothesized that during RM, a high Fio_2_ (1.0) leads to a higher risk for postoperative atelectasis in adults undergoing laparoscopic surgery, without benefiting oxygenation, than low Fio_2_ (0.4).

## Methods

### Design

This prospective, patient- and sonographer-blinded, single-center, parallel, randomized, controlled trial was approved by the Institutional Review Board of Seoul National University Hospital (No. 1903–137-1020, 22 April 2019) and registered at ClinicalTrials.gov (NCT03943433, 7 May 2019). The study was conducted in accordance with CONSORT guidelines. We enrolled adult patients scheduled to undergo elective laparoscopic gynecologic surgery or colorectal surgery in the Trendelenburg position from May to November 2019 after obtaining written informed consent. The inclusion criterion was adult patients aged 20–70 years with an American Society of Anesthesiologists physical status I–II. The exclusion criteria were patients with body mass index ≥35 kg m^− 2^, cardiovascular impairment, severe chronic obstructive pulmonary disease (preoperative forced expiratory volume in 1 s/forced vital capacity of 60% or lower) or emphysema, pneumothorax or bullae, previous lung resection surgery, and increased intracranial pressure. Some patients dropped out because of protocol violation, massive bleeding with hemodynamic compromise, or unexpected open conversion.

Patients were randomly assigned to two groups based on the applied Fio_2_ during RM, in a 1:1 ratio, by computer-generated randomization, using R software (version 3.5.1, R Foundation for Statistical Computing, Vienna, Austria). Allocation was concealed in an opaque envelope by an assistant not involved in the study and was delivered to the attending anesthesiologist before general anesthesia induction. The sonographer (BRK or HB) was completely blinded to the group assignment.

### Anesthesia and ventilator strategy

General anesthesia was induced according to the predetermined protocol with standard monitoring of pulse oximetry (Spo_2_), non-invasive blood pressure, electrocardiography, bispectral index (A-2000 XP; Aspect Medical Systems, Newton, MA), and end-tidal carbon dioxide concentration. After preoxygenation with 100% oxygen, propofol 1.5–2.0 mg kg^− 1^ was administered intravenously with a continuous target-controlled remifentanil infusion (Orchestra; Fresenius Kabi, Brézins, France). Rocuronium 0.6–0.8 mg kg^− 1^ was administered for neuromuscular blockade, and tracheal intubation was performed. General anesthesia was maintained with sevoflurane and remifentanil to maintain the bispectral index within 40–60. A radial arterial catheter was placed and connected to an arterial waveform analysis system (Flotrac; Edwards Lifesciences, Irvine, CA) for close monitoring of intraoperative hemodynamic changes derived from the RM, as a part of the institutional protocols.

Mechanical ventilation was maintained intraoperatively with the Fio_2_ at 0.4, tidal volume at 8 ml kg^− 1^ of ideal body weight, PEEP at 5 cmH_2_O, inspiration to expiration ratio of 1:2, and end-inspiration pause 10% at volume-controlled ventilation mode. Respiratory rate was adjusted to maintain partial pressure of arterial carbon dioxide at 35–45 mmHg. If the peak airway pressure exceeded 35 cmH_2_O, the tidal volume was decreased stepwise by 1 ml kg^− 1^ until the peak pressure was < 35 cmH_2_O.

At the end of the surgery, sugammadex 2–4 mg kg^− 1^ was administered after train-of-four count monitoring for reversal of neuromuscular blockade. The Fio_2_ was changed to 1.0 when the first spontaneous breathing was observed. After extubation, patients were transferred to the post-anesthesia care unit (PACU). Intravenous patient-controlled analgesia was routinely used for postoperative pain control. Patients were discharged from the PACU when they met the Modified Aldrete Score criteria [20].

### Lung ultrasound examination and RM strategy

**Fig. 1:**
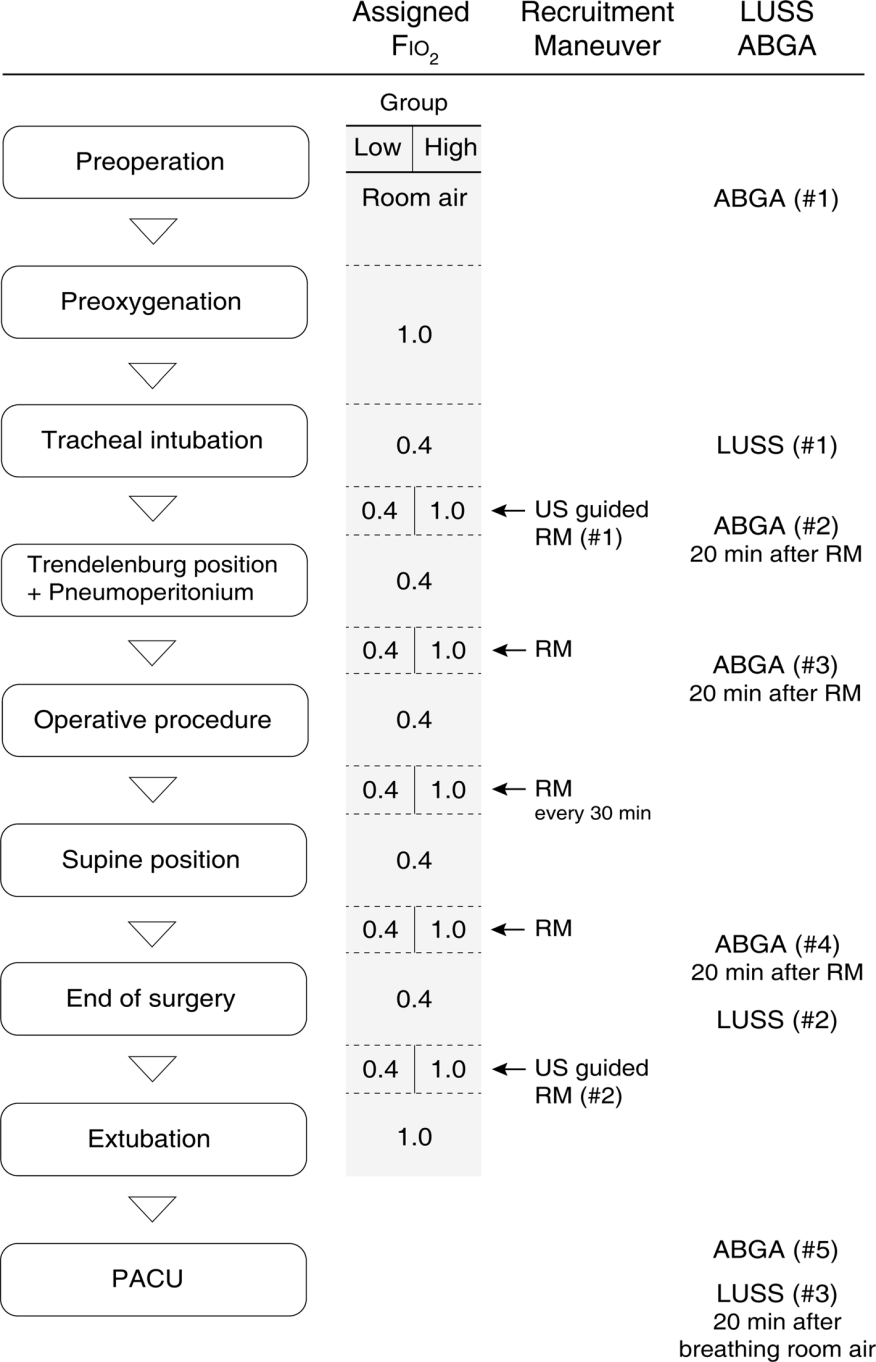
Experimental protocol during general anesthesia. LUSS, lung ultrasound score; ABGA, arterial blood gas analysis; US, ultrasound; RM, recruitment maneuver; PACU, post-anesthesia care unit

**Fig. 2:**
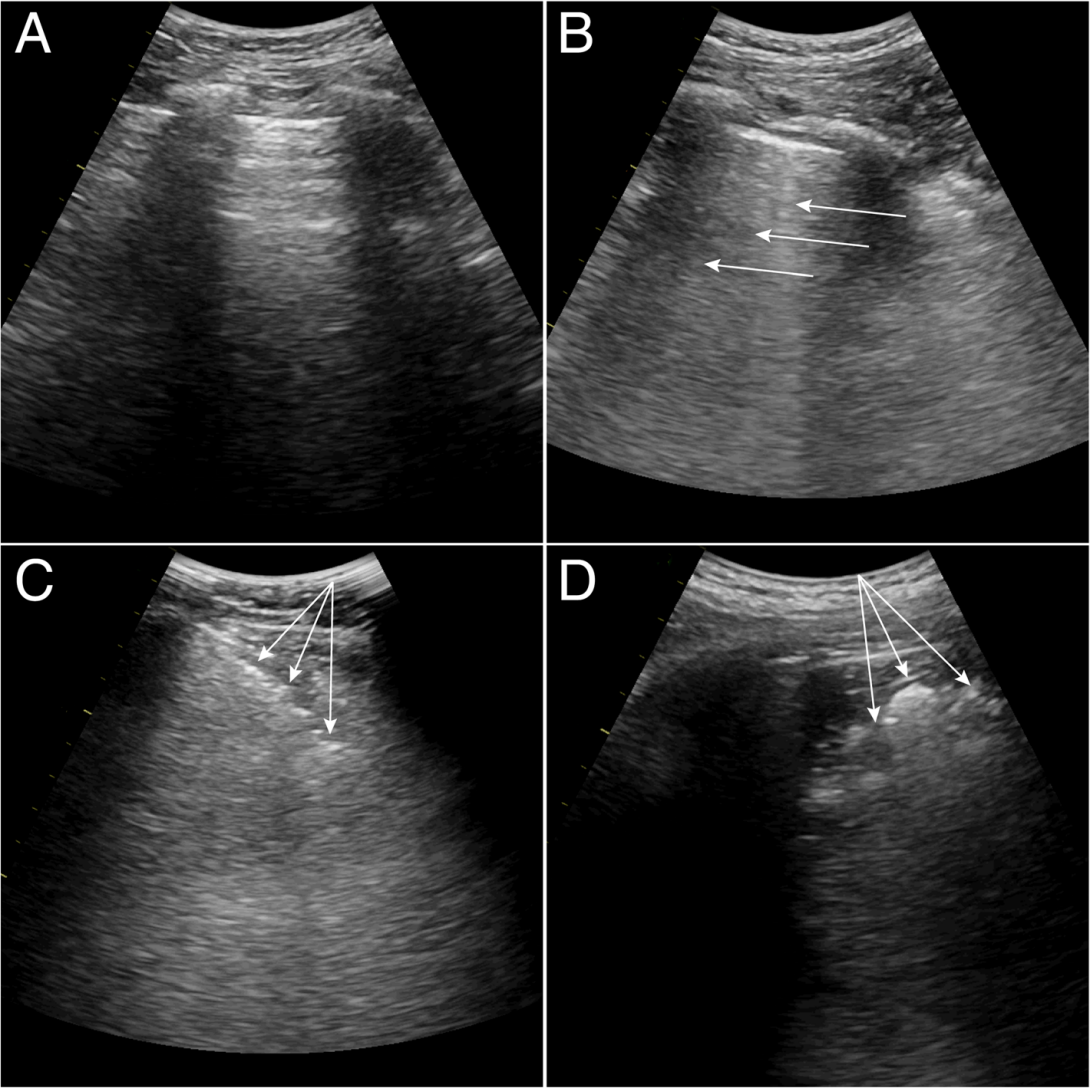
Lung ultrasound findings with different scores. Modified lung ultrasound scoring system in accordance with the method of Monastesse et al. (A) Normal pattern ‘bat-sign’ with A-lines parallel to the pleural line, score = 0; (B) More than three B lines arising from pleural line, score = 1; (C) Multiple subpleural consolidations separated by an irregular pleural line, score = 2; (D) Large-sized consolidation, score = 3. Each arrow indicates pathologic findings of each figure

Lung ultrasound examination was performed at three time-points: 1 min after starting mechanical ventilation, at the end of surgery (before emergence), and after breathing room air for 20 min at PACU (Fig. 1). Lung ultrasound was performed by two investigators (BRK and HB) blinded to the group assignment. Both investigators had performed more than 100 cases of lung ultrasound. The ultrasound was performed in the supine position using a Vivid-I ultrasound device (GE Healthcare, Chalfont St. Giles, Bucks, UK) and a convex probe, with a frequency of 2.5 MHz–7.5 MHz. All intercostal spaces were examined as previously described: each hemithorax was divided into six regions with three longitudinal lines (parasternal, anterior, and posterior axillary) and two axial lines (one above the diaphragm and another at 1 cm above the nipples) [15]. Each region was scored according to the modified LUSS system suggested by Monastesse et al., which showed sufficient sensitivity to detect loss of aeration during laparoscopic surgery [21]. The degree of deaeration was rated from 0 to 3 as follows (Fig. 2): 0, 0–2 B lines; 1, ≥3 B lines or 1 or multiple subpleural consolidations separated by a normal pleural line; 2, multiple coalescent B lines or multiple subpleural consolidations separated by a thickened or irregular pleural line; and 3, consolidation or small subpleural consolidation exceeding 1 × 2 cm in diameter [21]. The points for the 12 regions were summed for analysis. Furthermore, we defined substantial atelectasis as a score of 2 or 3 assigned to any region.

RM was performed after lung ultrasound examinations (twice) under real-time ultrasound guidance, with the probe placed at the region with the highest score. After setting the Fio_2_ (1.0 or 0.4) according to the assignment, continuous positive airway pressure was applied from 15 cmH_2_O in 5-cmH_2_O stepwise increments, up to the pressure at which no collapsed area was observed. The maximum continuous airway pressure applied during RM was 40 cmH_2_O. The applied pressure (opening pressure) and the duration of the RM were recorded. Additional intraoperative RM was performed at several time-points: at the time of Trendelenburg positioning and at every 30 min thereafter, and after a return to supine position after procedure completion (Fig. 1). Intraoperative RM was performed using the initial pressure and duration after adjustment of Fio_2_ according to the group assignment. The pre-designated Fio_2_ was applied only during the RM, after which it was maintained at 0.4 throughout mechanical ventilation in both groups.

### Outcomes

The primary outcome was the modified LUSS at surgery completion (before emergence), reflecting an aeration loss during general anesthesia. The secondary outcomes were the modified LUSS at PACU, substantial atelectasis observed on lung ultrasound, intraoperative and postoperative Pao_2_ to Fio_2_ ratios, and incidences of intraoperative desaturation (Spo_2_ < 95%), postoperative fever (body temperature > 38 °C during hospital stay), and postoperative pulmonary complications during hospital stay. Arterial blood samples were obtained 20 min after a change in position from supine to Trendelenburg and after breathing room air for 20 min at the PACU. Postoperative atelectasis, pneumonia, acute respiratory distress syndrome, and pulmonary aspiration data were collected by reviewing medical records. Their severity was evaluated based on previous consensus definitions for standardized perioperative pulmonary complications [22]. In our study, in-hospital pulmonary complications were atelectasis, pneumonia, acute respiratory distress syndrome and mild-to-severe pulmonary aspiration. Data on postoperative pulmonary complications were collected during the hospital stay. Additionally, data on age, height, weight, sex, type of operation, duration of anesthesia and surgery, pressure and duration of RM, and ventilator parameters were collected. Significant hemodynamic deterioration during RM (> 20% of baseline) was documented and treated with vasoactive drugs or crystalloid agents.

### Statistical analysis

In our pilot study on patients undergoing laparoscopic surgery in the Trendelenburg position (*n* = 10), the modified LUSS [mean (SD)] before and at the end of surgery were 3.88 (1.26) and 8.66 (2.82), respectively. Considering a 20% decrease in the modified LUSS in the low Fio_2_ group, we calculated that 44 patients would be needed in each group, with a type-I error risk of 0.05 and a power of 0.8 for two-tailed analysis.

Continuous variables were summarized as mean (SD) or median (interquartile range). The variables were analyzed using unpaired or paired *t*-tests and the Mann–Whitney U or Wilcoxon signed-rank tests, after assessing the normality of data distribution with the Shapiro–Wilk test. Number of patients (%) was compared using the chi-squared test or Fisher’s exact test. Statistical analyses were performed with R software (version 3.5.1, R Foundation for Statistical Computing, Vienna, Austria). For all analyses, *P* < 0.05 was statistically significant.

## Results

**Fig. 3:**
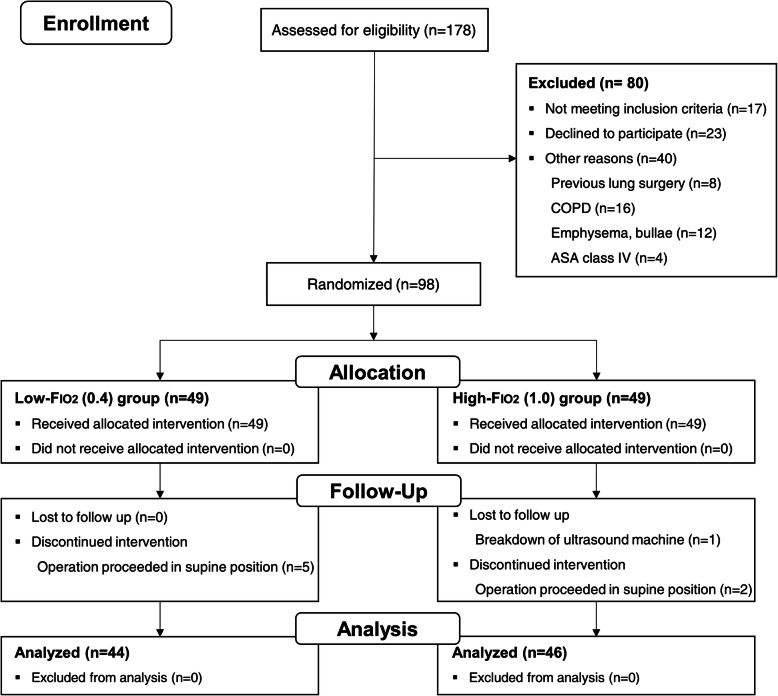
CONSORT diagram. COPD, chronic obstructive pulmonary disease; ASA, American Society of Anesthesiologists

One-hundred-and-seventy-eight patients scheduled to undergo laparoscopic surgery in the Trendelenburg position were assessed for eligibility. Among them, 98 patients met the inclusion criteria and were randomized to the low- (*n* = 49) or the high-Fio_2_ groups (n = 49). Five patients in the low-Fio_2_ and two patients in the high-Fio_2_ group dropped out owing to an intraoperative change to supine position. One patient was excluded owing to an ultrasound machine breakdown. Consequently, 44 and 46 patients in each group were analyzed, respectively (Fig. 3).

Participants’ baseline characteristics are summarized in Table 1. The groups did not differ in terms of patient characteristics or operational data. The modified LUSS are presented in Table 2. The baseline modified LUSS, measured at 1 min after anesthesia induction did not differ between the groups (*P* = 0.747). For the primary outcome, the modified LUSS at the end of surgery was significantly lower in the low-Fio_2_ group (median difference 5.0, 95% CI 3.0–7.0, *P* < 0.001). Moreover, the modified LUSS at 20 min after breathing room air at the PACU was significantly lower in the low-Fio_2_ group (*P* < 0.001). Substantial atelectasis at 1 min after starting mechanical ventilation was observed in 12 (27.3%) and 15 (32.6%) patients in the low- and high-Fio_2_ groups, respectively (*P* = 0.747). However, this was more frequently observed in the high-Fio_2_ after surgery completion (relative risk 1.77, 95% CI 1.27–2.47, *P* < 0.001) and at PACU (relative risk 1.73, 95%CI 1.26–2.38, *P* < 0.001).

**Table 1:** Characteristics of patients, surgery, and anesthesia

	Low-Fio_2_ group (*n* = 44)	High-Fio_2_ group (*n* = 46)	*P*-value
Age (year)	49.5 (43.0–59.0)	54.5 (43.0–61.0)	0.508
Female, n	32 (72.7)	28 (60.9)	0.332
Height (cm)	158.5 (154.7–165.5)	161.4 (156.0–170.0)	0.184
Weight (kg)	61.7 (54.0–68.9)	60.5 (54.0–70.0)	0.987
Predicted body weight (kg)	52.0 (48.0–59.5)	54.0 (48.0–66.0)	0.140
Body mass index (kg m^−2^)	23.7 (21.8–26.2)	24.1 (21.0–25.9)	0.617
ASA classification (I/II), n	31/13	24/22	0.118
**Comorbidity**			
Hypertension, n	9 (20.5)	11 (23.9)	0.888
Diabetes mellitus, n	3 (6.8)	7 (15.2)	0.351
Current Smoker, n	4 (9.1)	7 (15.2)	0.572
ARISCAT score	23 (8–26)	19 (7–26)	0.322
**Type of surgery**			0.406
Laparoscopic colorectal surgery, n	21 (47.7)	27 (58.7)	
Laparoscopic gynecologic surgery, n	23 (52.3)	19 (41.3)	
**Operative profiles**			
Duration of anesthesia (min)	147.5 (107.5–195.5)	170.0 (115.0–230.0)	0.109
Duration of surgery (min)	100.0 (70.0–140.0)	125.0 (85.0–180.0)	0.058
Duration of Trendelenburg position (min)	70.0 (46.5–100.5)	80.0 (56.0–142.0)	0.054
Intraoperative crystalloid administration (ml)	600.0 (500.0–875.0)	700.0 (400.0–1000.0)	0.484
Estimated blood loss (ml)	65.0 (40.0–112.5)	100.0 (50.0–200.0)	0.145
Urine output (ml)	130.0 (80.0–200.0) (*n* = 39)*	150.0 (85.0–265.0) (*n* = 43)*	0.111
Intraoperative inotropic requirement, n	20.0 (45.4)	28.0 (60.9)	0.356

Values are expressed as median (Interquartile range) or number (%). ASA, American Society of Anesthesiologists; ARISCAT, Assess Respiratory Risk in Surgical patients in Catalonia. *Urine output was measured in patients with Foley catheter

**Table 2:** Intraoperative and postoperative modified lung ultrasound scores

	Low-Fio_2_ group (n = 44)	High-Fio_2_ group (n = 46)	*P*-value
**Baseline, after intubation**			
Substantial atelectasis, n	12 (27.3%)	15 (32.6%)	0.747
Total modified LUSS	5.0 (3.0–8.0)	4.0 (4.0–6.0)	0.524
Anterior regions	0.0 (0.0–1.0)	0.0 (0.0–1.0)	0.538
Lateral regions	1.0 (0.0–2.5)	1.0 (0.0–2.0)	0.427
Posterior regions	4.0 (2.0–4.0)	3.0 (3.0–4.0)	0.839
**End of surgery, before extubation**			
Substantial atelectasis, n	21 (47.7%)	39 (84.8%)	< 0.001
Total modified LUSS	6.0 (4.5–9.0)	12.0 (9.0–14.0)	< 0.001
Anterior regions	0.0 (0.0–2.0)	2.0 (1.0–4.0)	< 0.001
Lateral regions	1.5 (0.0–2.5)	3.5 (2.0–4.0)	< 0.001
Posterior regions	4.0 (3.0–6.0)	6.0 (5.0–8.0)	< 0.001
**Post-anesthesia care unit, before discharge**			
Substantial atelectasis, n	22 (50.0%)	40 (87.0%)	< 0.001
Total modified LUSS	7.0 (5.0–10.0)	12.0 (10.0–16.0)	< 0.001
Anterior regions	1.0 (0.0–2.5)	3.0 (2.0–4.0)	< 0.001
Lateral regions	2.0 (1.0–3.5)	4.0 (2.0–5.0)	< 0.001
Posterior regions	4.0 (3.0–6.0)	7.0 (5.0–8.0)	< 0.001

Data are expressed as median (interquartile range), or number (%). Anterior, lateral, and posterior regions of the thorax were divided by the anterior and posterior axillary lines. LUSS, lung ultrasound score

The perioperative Pao_2_ to Fio_2_ ratio did not differ between the groups at any time-point (Table 3). The incidence of intraoperative desaturation and the lowest Spo_2_ value during anesthesia did not differ between the groups (*P* = 0.959 and *P* = 0.119, respectively) (Table 4). Hemodynamic and respiratory variables in the Trendelenburg position with capnoperitoneum are summarized in Table 4.

**Table 3:** Perioperative Pao_2_ to Fio_2_ ratio from arterial blood gas analysis

	Low-Fio_2_ group (n = 44)	High-Fio_2_ group (n = 46)	*P*-value
Baseline, preoperative	430.0 (385.0–492.5)	438.0 (370.0–485.0)	0.422
Intraoperative			
20 min after induction	490.0 (410.0–531.2)	437.5 (375.0–530.0)	0.364
20 min after Trendelenburg	405.0 (111.4)	408.6 (123.5)	0.884
20 min after supine	471.8 (117.4)	490.8 (142.2)	0.492
Post-anesthesia care unit, postoperative	457.5 (397.5–552.5)	455.0 (400.0–495.0)	0.448

Data are expressed as mean (standard deviation) or median (Interquartile range)

**Table 4:** Intraoperative and postoperative variables

	Low-Fio_2_ group (n = 44)	High-Fio_2_ group (*n* = 46)	*P*-value
**Hemodynamic variables during anesthesia**			
Heart rate (beats min^−1^)	62.2 (57.0–67.4)	62.5 (57.8–70.3)	0.214
Mean arterial pressure (mmHg)	88.8 (9.3)	87.8 (7.8)	0.586
Cardiac index (L min^−1^ m^−2^)	2.5 (2.2–3.3)	2.5 (2.1–3.0)	0.457
Stroke volume variation (%)	9.5 (3.9)	10.8 (3.6)	0.103
Mean Spo_2_ (%)	99.9 (99.5–100.0)	99.8 (99.1–100.0)	0.154
Lowest Spo_2_ (%)	99.0 (98.0–100.0)	98.0 (97.0–100.0)	0.119
Intraoperative desaturation (Spo_2_ < 95%), n	3 (6.8%)	2 (4.3%)	0.959
**Respiratory parameters during capnoperitoneum**			
Minute ventilation (L min^−1^)	6.3 (0.9)	6.4 (1.0)	0.563
Peak inspiratory pressure (cmH_2_O)	23.8 (3.6)	23.3 (3.1)	0.485
Static compliance (ml cmH_2_O^−1^)	29.7 (7.8)	30.1 (7.3)	0.776
**Postoperative outcome variables**			
Fever within postoperative 24 h (> 38.0 °C), n	6 (13.6%)	10 (21.7%)	0.317
Atelectasis on postoperative chest X-ray, n	4 (9.1%)	3 (6.5%)	0.710
Length of hospital stay (day)	3.5 (2.0–5.0)	5.0 (2.0–6.0)	0.096
In-hospital pulmonary complication, n	0 (0.0%)	0 (0.0%)	

Data are expressed as mean (standard deviation), median (interquartile range), or number (%)

The opening pressure for the RM varied from 25 to 40 cmH_2_O and was similar between groups (*P* = 0.773). For 38 patients in the low-Fio_2_ group (86.4%) and 40 patients in the high-Fio_2_ group (87.0), 30 cmH_2_O was used to resolve the atelectasis. An opening pressure of 35 cmH_2_O was needed for four (9.1%) and for five (10.9%) patients in the low-Fio_2_ and high-Fio_2_ groups, respectively. For one patient in each group, an opening pressure of 25 cmH_2_O was required. One patient in the low-Fio_2_ group required 40 cmH_2_O to restore all collapsed areas. Hemodynamic deterioration was observed in 21 (47.7%) and 20 (43.5%) patients during RM in the low- and high-Fio_2_ groups, respectively (*P* = 0.687).

No postoperative pulmonary complication was reported during hospital stay. (Table 4). Five (9.1%) and 3 (6.5%) patients showed subsegmental atelectasis on postoperative radiographs in the low- and high-Fio_2_ groups, respectively (*P* = 0.710). Postoperative fever (>38 °C) occurred in 17.8% of the study population, with a similar incidence between the 2 groups (*P* = 0.317).

## Discussion

This study evaluated the impact of Fio_2_ during RM on development of postoperative atelectasis, using lung ultrasound. The postoperative modified LUSS was higher in the high-Fio_2_ group, indicating more severe aeration loss in this group. In addition, postoperative consolidation was more frequently observed in the high-Fio_2_ group, with no significant difference in the preoperative modified LUSS. Oxygenation was similar between groups at any time-point. These observations were consistent with our hypothesis that using a high Fio_2_ (1.0) during RM would not benefit oxygenation and lead to more postoperative atelectasis than using a low Fio_2_ (0.4).

High Fio_2_ is associated with the development of absorption atelectasis during general anesthesia [23, 24]. However, to the best of our knowledge, the impact of a temporary high Fio_2_ during RM on atelectasis development has not been investigated. In this study, patients assigned to the high-Fio_2_ group received RM with Fio_2_ 1.0, whereas those in the low-Fio_2_ group received RM with Fio_2_ 0.4. The Fio_2_ was uniformly maintained at 0.4 with 5-cmH_2_O PEEP during post-RM mechanical ventilation in both groups. A high oxygen concentration in the alveoli during RM was predicted to cause increased absorption atelectasis. Consequently, the postoperative modified LUSS was significantly lower in the low-Fio_2_, with the difference persisting in the PACU.

Using computed tomography, Rothen et al. demonstrated the progression of absorption atelectasis over time after RM in 12 patients, with an Fio_2_ of 0.4 or 1.0 during RM and thereafter [25]. Absorption atelectasis developed within 5 min in the Fio_2_ 1.0 group and after 40 min in the Fio_2_ 0.4 group. Although the impact of oxygen concentration was obvious, this and the present study differed in that the previous study applied the designated Fio_2_ not only during RM, but also during the rest of the study period. Additionally, Song et al. studied absorption atelectasis based on the Fio_2_ during mechanical ventilation, using lung ultrasound in children [14]. Although the Fio_2_ had no significant impact on the incidence of significant atelectasis (consolidation score ≥ 2), a high Fio_2_ led to higher consolidation and B-line scores. The study compared Fio_2_ of 0.3 and 0.6, which is a relatively small difference, and did not include laparoscopic surgeries in the Trendelenburg position, which may explain its discrepancy with our results. Recently, Cohen et al. showed that difference of Fio_2_ (0.3 vs. 0.8) throughout the surgery did not increase the risk of postoperative pulmonary complications [26], while we performed a randomized controlled trial to show that short exposure to high Fio_2_ (1.0) during the alveolar recruitment maneuver may affect postoperative atelectasis. According to Edmark et al., however, Fio_2_ 0.8 was reported as being of borderline importance as a cause of absorption atelectasis, and they further reported that absorption atelectasis occurred when exposed to Fio_2_ 1.0, even during short preoxygenation [23]. With respect to the diagnostic method, our study performed LUSS in the immediate postoperative period for all patients while Cohen et al. detected patients with pulmonary complications by reviewing diagnosis codes or events documented in the medical chart.

We observed no significant difference in the Pao_2_ to Fio_2_ ratio at any time-point. Recruitment of collapsed alveoli with high oxygen concentrations led to a rapid re-collapse of the inflated alveoli than benefiting oxygenation. In clinical practice, Fio_2_ may be increased during RM for rapid improvements in Spo_2_, in cases of desaturation during surgery. Nonetheless, we found that a high Fio_2_ during RM did not actually improve oxygenation, despite a transient, rapid increase in Spo_2_. A recent study of 32 patients undergoing laparoscopic cholecystectomy compared Pao_2_ levels after two times of intraoperative RM, with Fio_2_ 0.3 and Fio_2_ 1.0 [27]. Although the intraoperative Pao_2_ did not differ between the groups, it was significantly better in the Fio_2_ 0.3 group on postoperative blood gas analysis. This finding differed from that in our study because of possible differences in the mean operation time and the patients’ position. During surgery in a sitting position, such as laparoscopic cholecystectomy, the atelectasis may be more affected by Fio_2_ than other factors, compared to in surgery performed in a Trendelenburg position.

In our study, the overall intraoperative desaturation incidence was markedly lower than that in the study of Monastesse et al.; this could be mainly due to repetitive RM [defined as Spo_2_ < 95% vs. Spo_2_ < 94%; 5/90 (5.6%) vs. 4/29 (13.8%), excluding a case of endobronchial intubation] [21]. In our study, the Spo_2_ did not decrease below 90% in either group, and no patient required a rescue by a change in the Fio_2_ or PEEP. Furthermore, in-hospital pulmonary complications were absent in both groups. This may have been due to the inclusion of only patients with a low risk of pulmonary complications, along with repeated RM during mechanical ventilation. Postoperative fever (>38 °C) developed in a considerable number of patients in both groups. The length of hospital stay was non-significantly longer in the high-Fio_2_ group.

The postoperative modified LUSS in this study was similar to that in the study by Monastesse et al. [21]. In our study, the PACU score in the low-Fio_2_ group was lower and that of the high-Fio_2_ group was higher than in the previous study, although the mean values in both studies were similar. We also analyzed the incidence of substantial atelectasis, which was observed in > 80% of patients in the high-Fio_2_ group. A higher score and consolidation were mainly observed in the posterior (dependent) part of the thorax, which can be attributed to pneumoperitoneum and the Trendelenburg position. As all patients showed at least a single, small, subpleural consolidation after pneumoperitoneum in the study of Monastesse et al. [21], this incidence of substantial atelectasis is likely to be acceptable. Nonetheless, the substantial atelectasis observed in our study did not alter the clinical outcome.

Our study had several limitations. First, ultrasound is an operator-dependent imaging modality [28], and observed findings may vary based on the operator’s experience. However, the sonographers in our study were well-experienced in lung ultrasound examination, and therefore, operator-related variations were minimal. Second, since only patients with a low risk of pulmonary complications were included; therefore, our results cannot be extended to patients with lung disease. Moreover, clinical consequences of the atelectasis may not have been observed for the same reason. Third, the anesthesiologist who performed the RM was not blinded. However, the anesthesiologist performing lung ultrasound for outcome measurement was blinded to the Fio_2_ used for the RM. Fourth, there is a possibility of incomplete intraoperative recruitment with the opening pressure obtained in the supine state before surgical incision. The opening pressure was used as access to the dependent part of the thorax was limited during the surgery. Nevertheless, it was considered to be sufficiently effective because RM was mostly performed at a high pressure of ≥30 cmH_2_O. Fifth, we applied uniform PEEP of 5 cmH_2_O to all patients, not an individualized PEEP. After open up the lung with RM, sufficient level of PEEP is required to keep the lung free of collapse. However, identifying the optimal PEEP is another topic that should be further discussed. Lastly, the definition of substantial atelectasis was not validated by previous studies. Although previous studies have used lung ultrasound as a diagnostic tool for atelectasis [15, 19, 21, 29–31], the criteria for substantial atelectasis are yet to be established.

In conclusion, for patients undergoing laparoscopic surgery in the Trendelenburg position, a higher LUSS, reflecting a higher degree of absorption atelectasis, was observed when RM was performed with a high Fio_2_ (1.0) than with a low Fio_2_ (0.4). We also found that using a high Fio_2_ during RM yields no oxygenation benefit and may result in more atelectasis than when using low Fio_2_.

## Data Availability

The datasets used and/or analyzed during the current study are available from the corresponding author on reasonable request.

## Abbreviations

RM: Recruitment maneuver
PEEP: Positive-end expiratory pressure
LUSS: Lung ultrasound score
PACU: Post-anesthesia care unit
